# More Efficient Approach: Independent Diagnostic Value of Audiovisual Sexual Stimulation for Psychogenic Erectile Dysfunction

**DOI:** 10.1007/s10508-023-02763-8

**Published:** 2024-01-02

**Authors:** Zhiwei Liu, Tao Wu, Shanjin Ma, Lei Wang, Xiaoye Jiang, Wei Xue, Qisheng Tang, Keying Zhang, Shaojie Liu, Chao Xu, Yurui Chen, Yenong Zhou, Jianjun Ma

**Affiliations:** 1 Department of Urology, Tangdu Hospital, Air Force Medical University, No.1 Xinsi Road, Baqiao District, Xi’an, 710038 China; 2 Department of Urology, Xijing Hospital, Air Force Medical University, Xi’an, China; 3 Department of Urology, Air Force Medical Center of PLA, Beijing, China; 4 Department of Nursing, Xijing Hospital, Air Force Medical University, Xi’an, China

**Keywords:** Psychogenic erectile dysfunction, Audiovisual sexual stimulation, Nocturnal penile tumescence and rigidity, Erectile dysfunction, DSM-5

## Abstract

The diagnostic value of audiovisual sexual stimulation (AVSS) for psychogenic erectile dysfunction (ED) is still unclear. We investigated the independent diagnostic value and optimal cut-off parameter of AVSS for psychogenic ED. All participants had received the AVSS test and nocturnal penile tumescence and rigidity (NPTR) monitoring at least twice. ED patients were divided into psychogenic ED and organic ED according to NPTR examination. The diagnostic accuracy of AVSS parameters was evaluated with the receiver operating characteristic (ROC) curve, and the Youden index was employed to determine the optimal diagnostic cut-off values. A total of 346 patients with ED and 60 healthy men were included in this study, among which 162 and 184 cases of psychogenic and organic ED were identified based on NPTR, respectively. When comparing the two ED groups, the area under the curve (AUC) of AVSS parameters was 0.85–0.89. Six-selected AVSS parameters could precisely diagnose psychogenic ED, exhibiting increased diagnostic specificity compared with corresponding sensitivity. When comparing psychogenic ED with the control group, the AUC of the tumescence of the tip was superior to the AUC other parameters (0.81 vs. 0.58, 0.66, 0.59, 0.53, 0.68), and the best determined diagnostic cut-off value was the tumescence of the tip < 29.87%. Independent AVSS could diagnose psychogenic ED objectively and effectively, and its diagnostic value was highest when 1.50% ≤ tumescence of the tip < 29.87%.

## Introduction

Erectile dysfunction (ED) is defined as the inability to attain or maintain sufficient penile rigidity for sexual satisfaction (Droller et al., 1993). Although ED is not a fatal disease, it has a severe impact on male self-esteem and quality of sexual relationships. As many as 150 million men worldwide are estimated to be diagnosed with ED (Burnett et al., 2018), and the number of ED cases could reach 322 million by 2025 (Shamloul & Ghanem, 2013). Penile erection is a biopsychological process mediated by multi-coordinated systems including psychological, endocrine, vascular, and neurological processes (Andersson & Wagner, 1995). As a result, ED can be divided into psychogenic, organic, or a mixed subtype based on etiology, where psychogenic ED is induced by psycho-neurological factors instead of organic lesions (Hatzimouratidis et al., 2010). Long-term primary ED normally ascribes to psychogenic ED (Nguyen et al., 2017), which is becoming prevalent in the younger generation due to raising social pressures. Therapeutic interventions for ED largely depend on its etiology and severity (Elhanbly et al., 2018). Therefore, for early intervention, proposing novel approaches to effectively identify patients with psychogenic ED needs to be investigated.

Generally, penile erection is primary affected by organic factors rather than psychological factors during rapid eye movement sleep (Hirshkowitz & Schmidt, 2005). Based on this concept, the RigiScan device for monitoring nocturnal penile tumescence and rigidity (NPTR) was introduced for differential diagnosis of organic and psychogenic ED (Bradley et al., 1985; Hatzimouratidis et al., 2010). Normal NPTR indicates normal penile structure (Zou et al., 2019), and several guidelines recommend NPTR as a mainstay of assessing organic penile function (Burnett et al., 2018; Hatzimouratidis et al., 2010; Zhang et al., 2016). However, the accuracy of NPTR can be affected by sleep quality and it is also time-consuming. Additionally, being limited by methodology and detection principle, the degree of sexual arousal in patients with psychogenic ED is not adequately defined by NPTR. Consequently, it is difficult for NPTR to efficiently assess the degree of the condition.

For the etiology of psychogenic ED, it is essential to consider how psychological factors, such as sexual desire, affect the development of ED. Apart from psychometric questionnaires, a more objective means of assessing impaired penile erection is available. Audiovisual sexual stimulation (AVSS) along with a RigiScan device is regarded as a cost-effective and time-saving approach for psychogenic ED auxiliary diagnosis. Since AVSS mimics a male sexual response in the awake state by individual erotic video, it is conducive to investigating the degree of impaired sexual arousal. In theory, AVSS is more sensitive than NPTR in evaluating how psychology impacts erectile function. However, most previous studies have employed AVSS as a screening tool in ED, and its independent diagnostic value and optimal cut-off parameters for psychogenic ED are still unclear (Mizuno et al., 2004). In the present study, we hypothesized that independent AVSS would be an efficient means for psychological ED.

## Method

### Participants

Clinical data from a total of 346 patients with ED who met the inclusion criteria were retrieved from the medical records of the Department of Urology at Tangdu Hospital from December 2015 to March 2021. Then, we recruited 60 healthy men from July 2022 to December 2022 to compare the fidelity of both the NPTR and AVSS tests and their applicability to the ED groups. The duration of ED has been a controversial subject, as to whether it should be of more than 3 months or more than 6 months. In this study patients who suffered from ED for over 3 months and attempted intercourse at least once a week were selected with respect to the 2016 Guidelines from the Chinese Medicine Association of Andrology Committee (Zhang et al., 2016). The inclusion criteria included patients with heterosexual orientation, complete medical records and normal hormone levels, an International Index of Erectile Function-5 (IIEF-5) < 22, and who received an AVSS test and underwent NPTR monitoring at least twice. Exclusion criteria included patients with spinal cord injuries, concurrent neurologic disease, severe heart disease or penile fibrosis, ongoing treatment with phosphodiesterase-5 inhibitors (PDE-5i), and poor quality of rapid eye movement sleep.

### Measures and Procedure

Participants wore an audio-visual headset and were separately seated at comfortable tables in private examination rooms with strict control of light and sound. The tip and base of the penis were connected to a RigiScan Plus device to detect erectile events. A baseline was recorded for 15 min prior to stimulation. The rigidity and tumescence degree of the penis was evaluated for 60 min while the patients were stimulated with a pornographic video of their choice from our regularly updated library. Any other form of penile stimulation was forbidden during the AVSS testing session.

All participants underwent NPTR monitoring with the RigiScan plus device in the sleep unit at least twice after the AVSS test. The penis was connected to the detecting device as described in the AVSS test, and the NPTR data were analyzed. Factors that may influence a patients’ sleep was forbidden during the test, such as tea, caffeine, alcohol, drugs, smoking, etc. The normal erectile event was defined as at least a 60% penile tip rigidity lasting for at least 10 min during NPTR monitoring, which is consistent with the EAU guidelines. Patients were divided into psychogenic ED and organic ED according to the NPTR examination.

### Statistical Analysis

Total sample size was calculated using PASS 15 software. Normal distribution of variables was confirmed by the Kolmogorov–Smirnov test prior to statistical analysis. Kruskal–Wallis test were used to explore the differences of continuous variants between the three groups, then student's t-test or the Mann–Whitney test were used to pairwise comparisons. Categorical variants were evaluated using the chi-square test. The diagnostic accuracy of AVSS parameters was evaluated with ROC curve analysis and the Youden index was employed to determine the optimal diagnostic cut-off values. A *p* < .05 was considered statistically significant.

## Results

### Patients’ Demographic and Baseline Characteristics

A total of 346 ED patients and 60 healthy men were included in this study, among which 162 and 184 cases of psychogenic and organic ED patients were identified based on NPTR, respectively. Some basic characteristics and demographics were compared between the three groups. Variables including marital status, age, and a history of smoking were significantly lower in patients with psychogenic ED and the healthy control group than the patients with organic ED (*p* < .05, Table 1). Additionally, ED duration of the psychogenic ED group was shorter than that of the organic ED group (*p* < .001, Table 1).

**Table 1:** Subject demographic and baseline characteristics

Variables	Healthy control (*n* = 60)	Psychogenic ED (*n* = 162)	Organic ED (*n* = 184)	*p*-value
^a^Age (years)	29.00 (5.00)	30.00 (7.00)	33.00 (13.00)	*p* < .001
Marital status, n (%)	33 (55.00)	114 (70.40)	148 (80.40)	*p* < .001
Presence of children, n (%)	21 (35.00)	62 (38.30)	88 (47.80)	*p* = .098
Smoking, n (%)	12 (20.00)	51 (31.50)	77 (41.80)	*p* = .005
Alcohol use, n (%)	18 (30.00)	24 (14.80)	43 (23.40)	*p* = .026
Hypertension, n (%)	–	3 (1.90)	7 (3.80)	*p* = .347
Diabetes, n (%)	–	5 (3.10)	12 (6.50)	*p* = .212
Premature ejaculation, n (%)	–	64 (39.50)	57 (31.00)	*p* = .097
^a^ED duration (months)	–	6.00 (8.00)	12.00 (24.00)	*p* < .001

^a^Data are shown as median (interquartile range)

ED: erectile dysfunction

### Comparison Between Three Groups via Nocturnal Penile Tumescence and Rigidity

The duration of erectile episodes (DOEE), duration of tip rigidity over 60% (DOTR60), number of normal events (NONE), number of erectile episodes (NOEE), average event rigidity (AER) of tip and base, and ∆tumescence of the tip and base were increased in patients with psychogenic ED and the healthy control group, compared with the patients with organic ED (*p* < .001, Table 2). Moreover, NOEE, ∆tumescence of the tip and base in the healthy control group were higher than that of the psychogenic ED group (*p* = .001, *p* < .001,* p* = .014, Table 2).

**Table 2:** Comparison between three groups via nocturnal penile tumescence and rigidity

Parameters	Healthy controls	Psychogenic ED	Organic ED	*p*-value
DOEE (min)	91.13 (45.19)	75.75 (73.31)	17.25 (47.13)	*p* < .001
DOTR60 (min)	32.50 (19.06)	34.25 (37.63)	1.50 (7.00)	*p* < .001
NONE	1.00 (1.00)	1.00 (1.00)	0	*p* < .001
NOEE	5.00 (3.00)	4.00 (2.00)^**^	1.00 (2.00)	*p* < .001
AER of tip (%)	49.00 (14.00)	60.00 (35.25)	11.00 (38.75)	*p* < .001
AER of base (%)	62.50 (28.50)	72.50 (33.00)	23.00 (55.00)	*p* < .001
∆Tumescence of tip (%)	47.35 (20.79)	32.79 (15.35)^**^	16.67 (24.91)	*p* < .001
∆Tumescence of base (%)	42.15 (22.23)	33.98 (9.31)^*^	25.00 (33.98)	*p* < .001

*ED* erectile dysfunction, *DOEE* duration of erectile episodes, *DOTR60* duration of tip rigidity over 60%, *NONE* number of normal events, *NOEE* number of erectile episodes, *AER* average event rigidity

Data are shown as median (interquartile range). ∆Tumescence = (increased or maximum tumescence–minimum tumescence)/minimum tumescence

*p < .05 between Healthy controls and psychogenic ED, **p < .01 between Healthy controls and psychogenic ED

### Comparison Between Three Groups via Audiovisual Sexual Stimulation

The AVSS test detected erectile events in all healthy controls (100%), 135 patients with psychogenic ED (83.33%) and 12 patients with organic ED (6.52%). The DOEE, DOTR60, AER of the tip and base, and ∆tumescence of the tip and base were increased in the psychogenic ED group and healthy controls, compared with organic ED group (*p* < .001, Table 3). Moreover, ∆tumescence of the tip and base in the healthy control group were higher than that of patients with psychogenic ED (*p* < .001,* p* = .002, Table 3), and was consistent with the NPTR results mentioned above.

**Table 3:** Comparison between three groups via audiovisual sexual stimulation

Parameters	Healthy controls	Psychogenic ED	Organic ED	*p*-value
DOEE (min)	19.25 (21.38)	16.13 (18.12)	0 (0)	*p* < .001
DOTR60 (min)	0.50 (4.00)	4.50 (14.63)^**^	0 (0)	*p* < .001
AER of tip (%)	27.00 (17.75)	43.00 (59.75)	0 (0)	*p* < .001
AER of base (%)	50.00 (15.75)	49.00 (66.50)	0 (0)	*p* < .001
∆Tumescence of tip (%)	44.36 (20.05)	25.17 (18.45)^**^	0 (0)	*p* < .001
∆Tumescence of base (%)	31.25 (12.08)	26.24 (18.17)^**^	0 (0)	*p* < .001

*ED* erectile dysfunction, *DOEE* duration of erectile episodes, *DOTR60* duration of tip rigidity over 60%, *AER* average event rigidity

Data are shown as median (interquartile range). ∆Tumescence = (increased or maximum tumescence–minimum tumescence)/minimum tumescence

*p < .05 between Healthy controls and psychogenic ED, **p < .01 between Healthy controls and psychogenic ED

### Diagnostic Accuracy and Optimal Cut-off Values for Psychogenic Erectile Dysfunction by Comparing the Two Erectile Dysfunction Groups

**Fig. 1:**
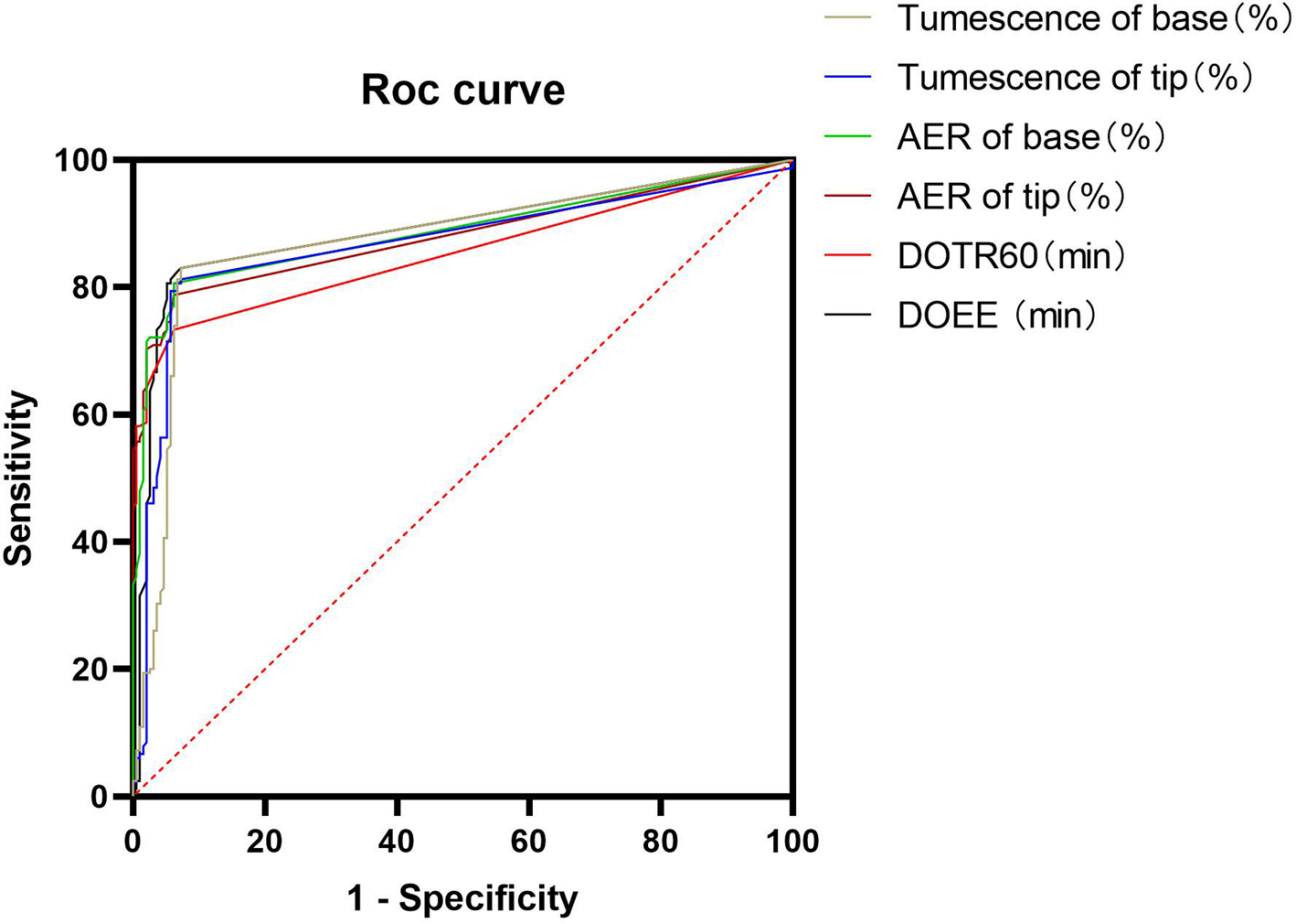
Receiver operating characteristic curve of audiovisual sexual stimulation parameters between two erectile dysfunction patient groups for psychogenic erectile dysfunction diagnosis. Six audiovisual sexual stimulation parameters could accurately diagnose psychogenic ED, significantly more accurate in AER of base and DOEE compared to other parameters. *ED: erectile dysfunction; DOEE: duration of erectile episodes; DOTR60: duration of tip rigidity over 60%; AER: average event rigidity

AVSS could accurately diagnose psychogenic ED regardless of parameters, and the area under the curve (AUC) of AVSS parameters ranged from 0.85 to 0.89. The AUC of DOTR60 was 0.85, while for both DOEE and AER of base it was 0.89 (Table 4 and Fig. 1), indicating that these AVSS parameters had significant diagnostic accuracy for psychogenic ED.

**Table 4:** Area under the curve for audiovisual sexual stimulation parameters in psychogenic erectile dysfunction diagnosis by comparing two erectile dysfunction patient groups

Parameters	AUC	95% confidence interval	
DOEE (min)	0.89	0.85	0.93
DOTR60 (min)	0.85	0.81	0.90
AER of tip (%)	0.88	0.84	0.92
AER of base (%)	0.89	0.85	0.93
∆Tumescence of tip (%)	0.87	0.82	0.91
∆Tumescence of base (%)	0.87	0.83	0.91

*AUC* area under the curve, *DOEE* duration of erectile episodes, *DOTR60* duration of tip rigidity over 60%, *AER* average event rigidity

∆Tumescence = (increased or maximum tumescence–minimum tumescence)/minimum tumescence

AVSS parameters exhibited diagnostic specificity compared with corresponding sensitivity (Table 5). Youden index was adopted to determine the cut-off values of different AVSS parameters for psychogenic ED diagnosis. As shown in Table 5, all AVSS provides a promising diagnostic indicator. The predicted best diagnostic cut-off values in AVSS parameters were DOEE ≥ 1.75 min, DOTR60 ≥ 0.25 min, AER of tip ≥ 0.50%, AER of base ≥ 0.50%, tumescence of the tip ≥ 1.50%, and tumescence of base ≥ 5.13%.

**Table 5:** Optimal cut-off values of audiovisual sexual stimulation for psychogenic erectile dysfunction by comparing two erectile dysfunction patient groups

Parameters	Sensitivity	Specificity	Youden index	Cut-off values
DOEE (min)	0.833	0.924	0.757	1.75
DOTR60 (min)	0.735	0.935	0.670	0.25
AER of tip (%)	0.790	0.935	0.725	0.50
AER of base (%)	0.809	0.935	0.744	0.50
∆Tumescence of tip (%)	0.815	0.924	0.739	1.50
∆Tumescence of base (%)	0.833	0.924	0.757	5.13

*DOEE* duration of erectile episodes, *DOTR60* duration of tip rigidity over 60%, *AER* average event rigidity

∆Tumescence = (increased or maximum tumescence–minimum tumescence)/minimum tumescence

### Diagnostic Accuracy and Optimal Cut-off Values for Psychogenic Erectile Dysfunction by Comparing Psychogenic Erectile Dysfunction with Healthy Controls

**Fig. 2:**
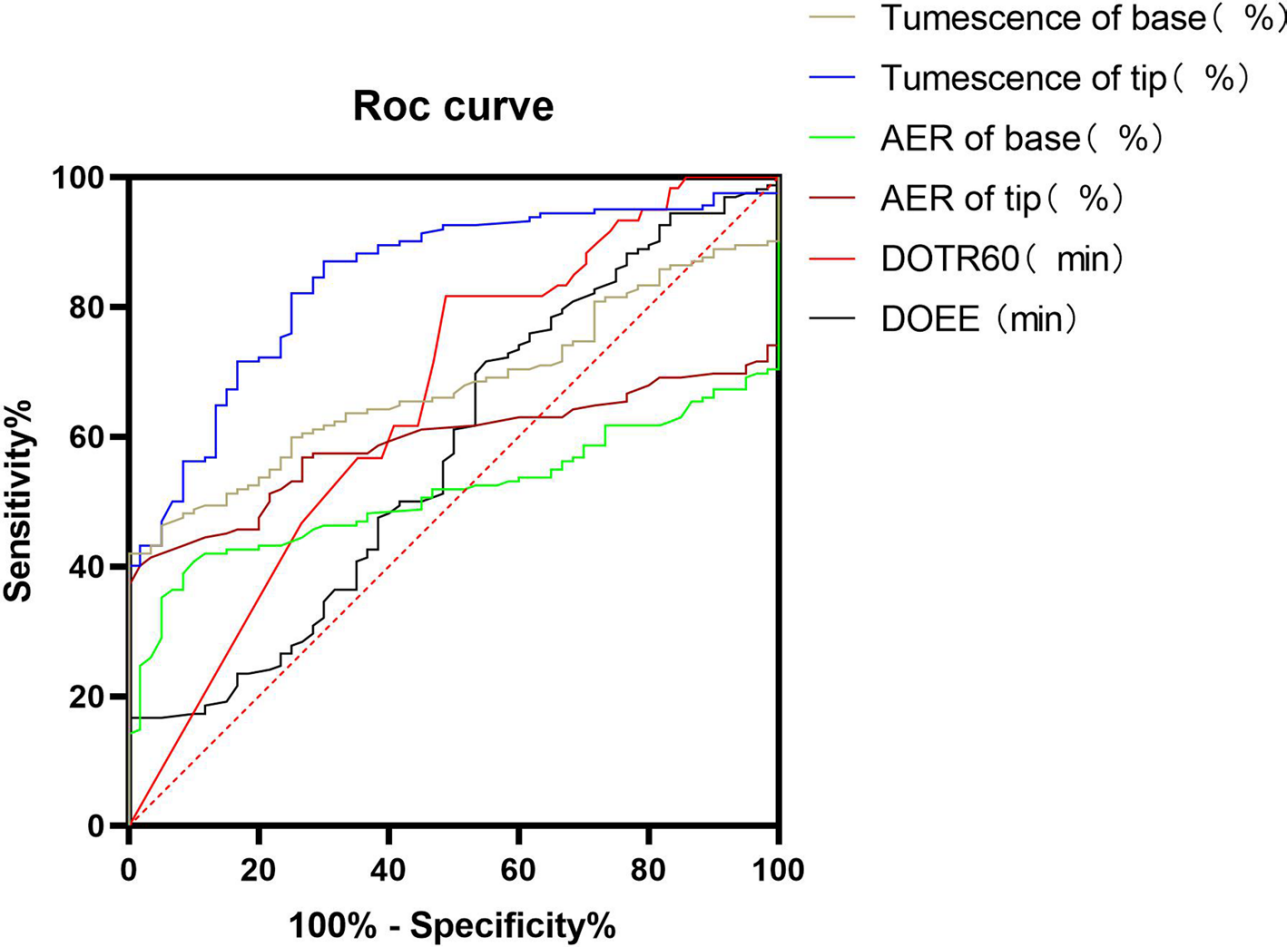
Receiver operating characteristic curve of audiovisual sexual stimulation parameters between psychogenic erectile dysfunction and healthy controls for psychogenic erectile dysfunction diagnosis. Six audiovisual sexual stimulation parameters had been shown above the figure, significantly more accurate in tumescence of tip compared to other parameters. *DOEE: duration of erectile episodes; DOTR60: duration of tip rigidity over 60%; AER: average event rigidity

The AUC of the tumescence of the tip was 0.81, which was higher than the other parameters (0.81 vs. 0.58, 0.66, 0.59, 0.53, 0.68) (Table 6 and Fig. 2), implying that tumescence of the tip had more significant diagnostic accuracy than other parameters of AVSS for psychogenic ED. As shown in Table 7, the predicted best diagnostic cut-off value was the tumescence of the tip < 29.87%, when compared to other AVSS parameters.

**Table 6:** Area under the curve for audiovisual sexual stimulation parameters in psychogenic erectile dysfunction diagnosis by comparing psychogenic erectile dysfunction with healthy controls

Parameters	AUC	95% confidence interval	
DOEE (min)	0.58	0.49	0.66
DOTR60 (min)	0.66	0.58	0.73
AER of tip (%)	0.59	0.52	0.66
AER of base (%)	0.53	0.46	0.60
∆Tumescence of tip (%)	0.81	0.75	0.86
∆Tumescence of base (%)	0.68	0.61	0.75

*AUC*:area under the curve, *DOEE* duration of erectile episodes, *DOTR60* duration of tip rigidity over 60%, *AER* average event rigidity

∆Tumescence = (increased or maximum tumescence–minimum tumescence)/minimum tumescence

**Table 7:** Optimal cut-off values of audiovisual sexual stimulation for psychogenic erectile dysfunction diagnosis by comparing psychogenic erectile dysfunction with healthy controls

Parameters	Sensitivity	Specificity	Youden index	Cut-off values
DOEE (min)	1	0.167	0.167	1.38
DOTR60 (min)	0	0.994	-0.006	40.75
AER of tip (%)	0.983	0.284	0.267	10.50
AER of base (%)	1	0.296	0.302	21.50
∆Tumescence of tip (%)	0.850	0.660	0.510	29.87
∆Tumescence of base (%)	1	0.420	0.420	23.03

*DOEE* duration of erectile episodes, *DOTR60* duration of tip rigidity over 60%, *AER* average event rigidity

∆Tumescence = (increased or maximum tumescence–minimum tumescence)/minimum tumescence

## Discussion

In this study, an independent AVSS test could predict psychogenic ED objectively and effectively. The results from the baseline characteristics between three the groups demonstrated that ED duration in patients with psychogenic ED tends to be less than that in patients with organic ED. Patients in the psychogenic ED group were generally younger than patients with organic ED, which is consistent with the awareness that young patients seem to be more susceptible to psychological factors. As first described in the 1970s, sexual stimulation and sexual desire play very important roles in a couples’ intercourse life (Masters, 1970). During the past decades, psychogenic ED was predominantly or exclusively ascribed to psychological or interpersonal factors (Rosen, 2001). Neuroimaging has confirmed that psychogenic ED can also be attributed to abnormalities in the central nervous system, characterized by less responsive activity in cerebral circuits during sexual arousal (Cera et al., 2012; Chen et al., 2017). According to Basson, most patients with ED have long-term concerns or confusion about erectile function before ED itself (Basson, 2015). Furthermore, for different stages of sexual response, especially the desired stage, a stable brain activation model has been established (Ruesink & Georgiadis, 2017). Since hypoactive sexual desire can contribute to the occurrence and development of psychogenic ED (Berger et al., 2016; Corona et al., 2016; Rastrelli et al., 2018), arousing sexual desire was proposed as an effective diagnostic and therapeutic strategy for psychogenic ED. Based on this theory, the AVSS test imitating sexual arousal in the awake state has been adopted to determine the severity of psychogenic ED (Cera et al., 2014). Despite classical NPTR monitoring in the differential diagnosis of organic and psychogenic ED (Zou et al., 2019), the AVSS test in hierarchical diagnosis and medication guidance of psychogenic ED cannot be replaced. However, it is unclear whether the AVSS test can determine the degree of impaired sexual desire, and its value and accuracy for psychogenic ED remains a gray area.

In general, the AVSS is regarded as an auxiliary diagnostic test for psychogenic ED and can be combined with intra-cavernous injection (ICI), pharmaco-penile duplex ultrasound (PPDU), or PDE-5i to differentiate psychogenic ED and vascular ED (Montorsi et al., 1996; Tang et al., 2013). These combined procedures improve the diagnostic accuracy for ED (Carneiro et al., 2020). As reported, the sensitivity and specialty of AVSS with PDE-5i administration in psychogenic ED diagnosis was increased to 87.7% and 93.4%, respectively (Wang et al., 2018). AVSS combined with ICI and PDDU achieved similar diagnostic values (Ardicoglu et al., 2005). These combined strategies are used in patients after radical prostatectomy or spinal injury, since fear of ICI and the potential contraindication of secondary diseases restrict their acceptance and widespread use (Mulhall et al., 1999). Additionally, combined strategies may mask organic factors, especially considering how one cannot exclude neurological or endocrine factors from psychogenic ED. Our previous study had demonstrated that AVSS parameters were correlated with ED treatment efficacy (Liu et al., 2022). Therefore, using AVSS alone with appropriate cut-off parameters might provide a cost-effective, objective, and efficient method for psychogenic ED diagnosis.

By comparing the two ED groups, we found that both AVSS and NPTR could identify patients with psychogenic ED from the patients with organic ED. Six select AVSS parameters could precisely diagnose psychogenic ED with excellent AUC (> 0.85), among which AER of base and DOEE had the highest diagnostic accuracy. These AVSS parameters exhibited increased diagnostic specificity compared with corresponding sensitivity, which is consistent with a previous report (Mizuno et al., 2004). Though AVSS could precisely diagnose psychogenic ED, its limitations should not be overlooked. When patients are repeatedly exposed to the same audiovisual stimulus, the AVSS-mediated diagnostic result might be offset (Kim et al., 1998). Other factors, including frequent pornographic viewing (Kraus et al., 2017) and sexual orientation (Rieger et al., 2005) maybe having an impact of results. Accordingly, erectile events were not triggered in 27 false negative patients in the psychogenic ED group during the AVSS test. Additionally, 12 patients had erectile events in organic ED group during the AVSS test, which might be due to two factors: Unsatisfactory rapid eye movement sleep quality can induce a false positive diagnosis by NPTR, and the increased diagnostic specificity guaranteed a true positive diagnosis by AVSS. The lack of psychometric tests to evaluate these patients may also affect the results of this study.

By comparing psychogenic ED and healthy controls, we found that the tumescence of the tip had the highest predictive value to distinguish psychogenic ED from healthy controls, which was mentioned sporadically during the last decades. Traditionally, several studies suggested that psychogenic ED was consistent with healthy people in NPTR variables. Our study had found the tumescence of the tip and base of NPTR parameters in the healthy control group was higher than that of the patients with psychogenic ED. Furthermore, it was interesting that similar results occurred in AVSS test, which may indicate these two parameters are more susceptible to psychogenic factors. However, the mechanism or underlying causes of such a result is still unclear.

Overall, according to this study, independent AVSS could precisely predict psychogenic ED patients, especially tumescence in the penile tip. Noticeably, the sample size of the present study was limited, and outcome bias could not be completely excluded. Therefore, the next step of the study is to validate the results with a larger sample or multicenter population.

### Conclusions

In conclusion, independent AVSS could diagnose psychogenic ED objectively and effectively, and its diagnostic value is highest when 1.50% ≤ tumescence of the tip < 29.87%.
